# Stock Market Volatility and Return Analysis: A Systematic Literature Review

**DOI:** 10.3390/e22050522

**Published:** 2020-05-04

**Authors:** Roni Bhowmik, Shouyang Wang

**Affiliations:** 1 School of Economics and Management, Jiujiang University, Jiujiang 322227, China; 2 Department of Business Administration, Daffodil International University, Dhaka 1207, Bangladesh; 3 Academy of Mathematics and Systems Science, Chinese Academy of Sciences, Beijing 100080, China; sywang@amss.ac.cn

**Keywords:** stock returns, volatility, GARCH family model, complexity in market volatility forecasting

## Abstract

In the field of business research method, a literature review is more relevant than ever. Even though there has been lack of integrity and inflexibility in traditional literature reviews with questions being raised about the quality and trustworthiness of these types of reviews. This research provides a literature review using a systematic database to examine and cross-reference snowballing. In this paper, previous studies featuring a generalized autoregressive conditional heteroskedastic (GARCH) family-based model stock market return and volatility have also been reviewed. The stock market plays a pivotal role in today’s world economic activities, named a “barometer” and “alarm” for economic and financial activities in a country or region. In order to prevent uncertainty and risk in the stock market, it is particularly important to measure effectively the volatility of stock index returns. However, the main purpose of this review is to examine effective GARCH models recommended for performing market returns and volatilities analysis. The secondary purpose of this review study is to conduct a content analysis of return and volatility literature reviews over a period of 12 years (2008–2019) and in 50 different papers. The study found that there has been a significant change in research work within the past 10 years and most of researchers have worked for developing stock markets.

## 1. Introduction

In the context of economic globalization, especially after the impact of the contemporary international financial crisis, the stock market has experienced unprecedented fluctuations. This volatility increases the uncertainty and risk of the stock market and is detrimental to the normal operation of the stock market. To reduce this uncertainty, it is particularly important to measure accurately the volatility of stock index returns. At the same time, due to the important position of the stock market in the global economy, the beneficial development of the stock market has become the focus. Therefore, the knowledge of theoretical and literature significance of volatility are needed to measure the volatility of stock index returns.

Volatility is a hot issue in economic and financial research. Volatility is one of the most important characteristics of financial markets. It is directly related to market uncertainty and affects the investment behavior of enterprises and individuals. A study of the volatility of financial asset returns is also one of the core issues in modern financial research and this volatility is often described and measured by the variance of the rate of return. However, forecasting perfect market volatility is difficult work and despite the availability of various models and techniques, not all of them work equally for all stock markets. It is for this reason that researchers and financial analysts face such a complexity in market returns and volatilities forecasting.

The traditional econometric model often assumes that the variance is constant, that is, the variance is kept constant at different times. An accurate measurement of the rate of return’s fluctuation is directly related to the correctness of portfolio selection, the effectiveness of risk management, and the rationality of asset pricing. However, with the development of financial theory and the deepening of empirical research, it was found that this assumption is not reasonable. Additionally, the volatility of asset prices is one of the most puzzling phenomena in financial economics. It is a great challenge for investors to get a pure understanding of volatility.

A literature reviews act as a significant part of all kinds of research work. Literature reviews serve as a foundation for knowledge progress, make guidelines for plan and practice, provide grounds of an effect, and, if well guided, have the capacity to create new ideas and directions for a particular area [1]. Similarly, they carry out as the basis for future research and theory work. This paper conducts a literature review of stock returns and volatility analysis based on generalized autoregressive conditional heteroskedastic (GARCH) family models. Volatility refers to the degree of dispersion of random variables.

Financial market volatility is mainly reflected in the deviation of the expected future value of assets. The possibility, that is, volatility, represents the uncertainty of the future price of an asset. This uncertainty is usually characterized by variance or standard deviation. There are currently two main explanations in the academic world for the relationship between these two: The leverage effect and the volatility feedback hypothesis. Leverage often means that unfavorable news appears, stock price falls, leading to an increase in the leverage factor, and thus the degree of stock volatility increases. Conversely, the degree of volatility weakens; volatility feedback can be simply described as unpredictable stock volatility that will inevitably lead to higher risk in the future.

There are many factors that affect price movements in the stock market. Firstly, there is the impact of monetary policy on the stock market, which is extremely substantial. If a loose monetary policy is implemented in a year, the probability of a stock market index rise will increase. On the other hand, if a relatively tight monetary policy is implemented in a year, the probability of a stock market index decline will increase. Secondly, there is the impact of interest rate liberalization on risk-free interest rates. Looking at the major global capital markets, the change in risk-free interest rates has a greater correlation with the current stock market. In general, when interest rates continue to rise, the risk-free interest rate will rise, and the cost of capital invested in the stock market will rise simultaneously. As a result, the economy is expected to gradually pick up during the release of the reform dividend, and the stock market is expected to achieve a higher return on investment.

Volatility is the tendency for prices to change unexpectedly [2], however, all kinds of volatility is not bad. At the same time, financial market volatility has also a direct impact on macroeconomic and financial stability. Important economic risk factors are generally highly valued by governments around the world. Therefore, research on the volatility of financial markets has always been the focus of financial economists and financial practitioners. Nowadays, a large part of the literature has studied some characteristics of the stock market, such as the leverage effect of volatility, the short-term memory of volatility, and the GARCH effect, etc., but some researchers show that when adopting short-term memory by the GARCH model, there is usually a confusing phenomenon, as the sampling interval tends to zero. The characterization of the tail of the yield generally assumes an ideal situation, that is, obeys the normal distribution, but this perfect situation is usually not established.

Researchers have proposed different distributed models in order to better describe the thick tail of the daily rate of return. Engle [3] first proposed an autoregressive conditional heteroscedasticity model (ARCH model) to characterize some possible correlations of the conditional variance of the prediction error. Bollerslev [4] has been extended it to form a generalized autoregressive conditional heteroskedastic model (GARCH model). Later, the GARCH model rapidly expanded and a GARCH family model was created.

When employing GARCH family models to analyze and forecast return volatility, selection of input variables for forecasting is crucial as the appropriate and essential condition will be given for the method to have a stationary solution and perfect matching [5]. It has been shown in several findings that the unchanged model can produce suggestively different results when it is consumed with different inputs. Thus, another key purpose of this literature review is to observe studies which use directional prediction accuracy model as a yardstick from a realistic point of understanding and has the core objective of the forecast of financial time series in stock market return. Researchers estimate little forecast error, namely measured as mean absolute deviation (MAD), root mean squared error (RMSE), mean absolute error (MAE), and mean squared error (MSE) which do not essentially interpret into capital gain [6,7]. Some others mention that the predictions are not required to be precise in terms of NMSE (normalized mean squared error) [8]. It means that finding the low rate of root mean squared error does not feed high returns, in another words, the relationship is not linear between two.

In this manuscript, it is proposed to categorize the studies not only by their model selection standards but also for the inputs used for the return volatility as well as how precise it is spending them in terms of return directions. In this investigation, the authors repute studies which use percentage of success trades benchmark procedures for analyzing the researchers’ proposed models. From this theme, this study’s authentic approach is compared with earlier models in the literature review for input variables used for forecasting volatility and how precise they are in analyzing the direction of the related time series. There are other review studies on return and volatility analysis and GARCH-family based financial forecasting methods done by a number of researchers [9,10,11,12,13]. Consequently, the aim of this manuscript is to put forward the importance of sufficient and necessary conditions for model selection and contribute for the better understanding of academic researchers and financial practitioners.

Systematic reviews have most notable been expanded by medical science as a way to synthesize research recognition in a systematic, transparent, and reproducible process. Despite the opportunity of this technique, its exercise has not been overly widespread in business research, but it is expanding day by day. In this paper, the authors have used the systematic review process because the target of a systematic review is to determine all empirical indication that fits the pre-decided inclusion criteria or standard of response to a certain research question. Researchers proved that GARCH is the most suitable model to use when one has to analysis the volatility of the returns of stocks with big volumes of observations [3,4,6,9,13]. Researchers observe keenly all the selected literature to answer the following research question: What are the effective GARCH models to recommend for performing market volatility and return analysis?

The main contribution of this paper is found in the following four aspects: (1) The best GARCH models can be recommended for stock market returns and volatilities evaluation. (2) The manuscript considers recent papers, 2008 to 2019, which have not been covered in previous studies. (3) In this study, both qualitative and quantitative processes have been used to examine the literature involving stock returns and volatilities. (4) The manuscript provides a study based on journals that will help academics and researchers recognize important journals that they can denote for a literature review, recognize factors motivating analysis stock returns and volatilities, and can publish their worth study manuscripts.

## 2. Methodology

**Figure 1:**
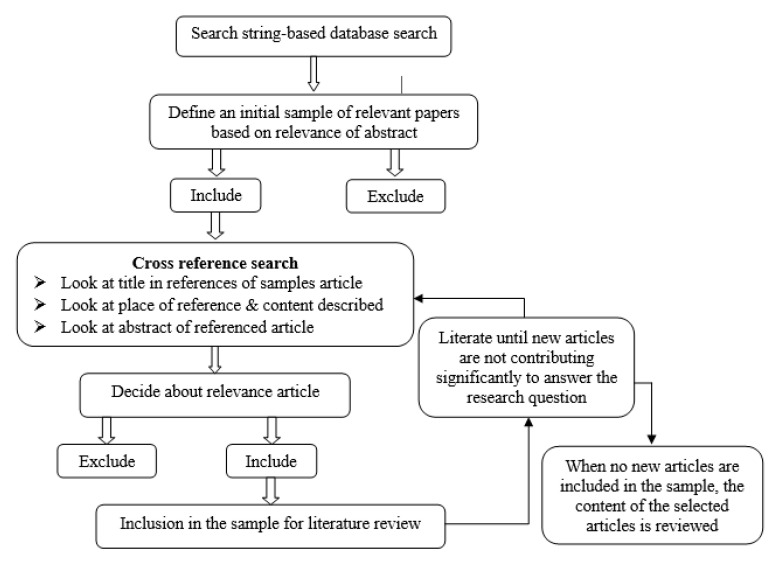
Literature review method.

A systematic literature examination of databases should recognize as complete a list as possible of relevant literature while keeping the number of irrelevant knocks small. The study is conducted by a systematic based literature review, following suggestions from scholars [14,15]. This manuscript was led by a systematic database search, surveyed by cross-reference snowballing, as demonstrated in Figure 1, which was adapted from Geissdoerfer et al. [16]. Two databases were selected for the literature search: Scopus and Web-of-Science. These databases were preferred as they have some major depositories of research and are usually used in literature reviews for business research [17].

At first stage, a systematic literature search is managed. The keywords that were too broad or likely to be recognized in literature-related keywords with other research areas are specified below. As shown in Table 1, the search string “market return” in ‘Title‘ respectively “stock market return”, “stock market volatility”, “stock market return volatility”, “GARCH family model* for stock return”, “forecasting stock return”, and GARCH model*, “financial market return and volatility” in ‘Topic’ separately ‘Article title, Abstract, Keywords’ were used to search for reviews of articles in English on the Elsevier Scopus and Thomson Reuters Web-of-Science databases. The asterisk (*) is a commonly used wildcard symbol that broadens a search by finding words that start with the same letters.

At second stage, suitable cross-references were recognized in this primary sample by first examining the publications’ title in the reference portion and their context and cited content in the text. The abstracts of the recognized further publications were examined to determine whether the paper was appropriate or not. Appropriate references were consequently added to the sample and analogously scanned for appropriate cross-references. This method was continual until no additional appropriate cross-references could be recognized.

At the third stage, the ultimate sample was assimilated, synthesized, and compiled into the literature review presented in the subsequent section. The method was revised a few days before the submission.

Additionally, the list of affiliation criteria in Table 2, which is formed on discussions of the authors, with the summaries of all research papers were independently checked in a blind system method. Evaluations were established on the content of the abstract, with any extra information unseen, and were comprehensive rather than exclusive. In order to check for inter-coder dependability, an initial sample of 30 abstracts were studied for affiliation by the authors. If the abstract was not satisfactorily enough, the whole paper was studied. Simply, 4.61 percent of the abstract resulted in variance between the researchers. The above-mentioned stages reduced the subsequent number of full papers for examination and synthesis to 50. In order to recognize magnitudes, backgrounds, and moderators, these residual research papers were reviewed in two rounds of reading.

## 3. Review of Different Studies

In this paper, a large amount of articles were studied but only a few were well thought out to gather the quality developed earlier. For every published article, three groups were specified. Those groups were considered as index and forecast time period, input elements, econometric models, and study results. The first group namely “index and forecast time period with input elements” was considered since market situation like emerging, frontier, and developed markets which are important parameters of forecast and also the length of evaluation is a necessary characteristic for examining the robustness of the model. Furthermore, input elements are comparatively essential parameters for a forecast model because the analytical and diagnostic ability of the model is mainly supported on the inputs that a variable uses. In the second group, “model” was considered forecast models proposed by authors and other models for assessment. The last group is important to our examination for comparing studies in relationships of proper guiding return and volatility, acquired by using recommended estimate models, named the “study results” group.

Measuring the stock market volatility is an incredibly complex job for researchers. Since volatility tends to cluster, if today’s volatility is high, it is likely to be high tomorrow but they have also had an attractive high hit rate with major disasters [4,7,11,12]. GARCH models have a strong background, recently having crossed 30 years of the fast progress of GARCH-type models for investigating the volatility of market data. Literature of eligible papers were clustered in two sub groups, the first group containing GARCH and its variations model, and the second group containing bivariate and other multivariate GARCH models, summarized in a table format for future studies. Table 3 explains the review of GARCH and its variations models. The univariate GARCH model is for a single time series. It is a statistical model that is used to analyze a number of different kinds of financial data. Financial institutions and researchers usually use this model to estimate the volatility of returns for stocks, bonds, and market indices. In the GARCH model, current volatility is influenced by past innovation to volatility. GARCH models are used to model for forecast volatility of one time series. The most widely used GARCH form is GARCH (1, 1) and this has some extensions.

In a simple GARCH model, the squared volatility $\sigma_{t}^{2}$ is allowed to change on previous squared volatilities, as well as previous squared values of the process. The conditional variance satisfies the following form: $\sigma_{t}^{2}=\alpha_{0}+\alpha_{1}\epsilon_{t-1}^{2}+\ldots +\alpha_{q}\epsilon_{t-q}^{2}+\beta_{1}\sigma_{t-1}^{2}+\ldots +\beta_{p}\sigma_{t-p}^{2}$ where, $\alpha_{i}>0$ and $\beta_{i}>0$. For the GARCH model, residuals’ lags can substitute by a limited number of lags of conditional variances, which abridges the lag structure and in addition the estimation method of coefficients. The most often used GARCH model is the GARCH (1, 1) model. The GARCH (1, 1) process is a covariance-stationary white noise process if and only if $\alpha_{1}+\beta <1$. The variance of the covariance-stationary process is given by $\alpha_{1}\;/\;\left(1-\alpha_{1}-\beta \right)$. It specifies that $\sigma_{n}^{2}\;\;$is based on the most recent observation of $\varphi_{t}^{2}\;$and the most recent variance rate $\sigma_{n-1}^{2}$. The GARCH (1, 1) model can be written as $\sigma_{n}^{2}=\omega +\alpha \varphi_{n-1}^{2}+\beta \sigma_{n-1}^{2}$ and this is usually used for the estimation of parameters in the univariate case.

Though, GARCH model is not a complete model, and thus could be developed, these developments are detected in the form of the alphabet soup that uses GARCH as its key component. There are various additions of the standard GARCH family models. Nonlinear GARCH (NGARCH) was proposed by Engle and Ng [18]. The conditional covariance equation is in the form: $\sigma_{t}^{2}=\gamma +\alpha {\left(\varepsilon_{t-1}-\vartheta \sigma_{t-1}\;\right)}^{2}+\beta \sigma_{t-1}^{2}$, where α, β, γ>0. The integrated GARCH (IGARCH) is a restricted version of the GARCH model, where the sum of all the parameters sum up to one and this model was introduced by Engle and Bollerslev [19]. Its phenomenon might be caused by random level shifts in volatility. The simple GARCH model fails in describing the “leverage effects” which are detected in the financial time series data. The exponential GARCH (EGARCH) introduced by Nelson [5] is to model the logarithm of the variance rather than the level and this model accounts for an asymmetric response to a shock. The GARCH-in-mean (GARCH-M) model adds a heteroskedasticity term into the mean equation and was introduced by Engle et al. [20]. The quadratic GARCH (QGARCH) model can handle asymmetric effects of positive and negative shocks and this model was introduced by Sentana [21]. The Glosten-Jagannathan-Runkle GARCH (GJR-GARCH) model was introduced by Glosten et al. [22], its opposite effects of negative and positive shocks taking into account the leverage fact. The threshold GARCH (TGARCH) model was introduced by Zakoian [23], this model is also commonly used to handle leverage effects of good news and bad news on volatility. The family GARCH (FGARCH) model was introduced by Hentschel [24] and is an omnibus model that is a mix of other symmetric or asymmetric GARCH models. The COGARCH model was introduced by Klüppelberg et al. [25] and is actually the stochastic volatility model, being an extension of the GARCH time series concept to continuous time. The power-transformed and threshold GARCH (PTTGARCH) model was introduced by Pan et al. [26], this model is a very flexible model and, under certain conditions, includes several ARCH/GARCH models.

**Table 3:** Different literature studies based on generalized autoregressive conditional heteroskedastic (GARCH) and its variations models.

Authors	Data Set	Econometric Models	Study Results
**Alberg et al.** [27]	Daily returns data, TASE indices, the TA25 index period October 1992 to May 2005 and TA100 index period July 1997 to May 2005	GARCH, EGARCH, and APARCH model	Findings suggest that one can improve overall estimation by using the asymmetric GARCH model and the EGARCH model is a better predictor than the other asymmetric models.
**Olowe** [28]	Daily returns over the period January 2004 to March 2009	EGARCH in mean model	Nigerian stock market returns show that volatility is persistent and there is a leverage effect. The study found little evidence open the relationship between stock returns and risk as measures by its aim volatility.
**Girard & Omran** [29]	Examine the interaction of volatility and volume in 79 traded companies in Cairo and Alexandria Stock Exchange	GARCH model	They found that information size and direction have a negligible effect on conditional volatility and, as a result, the presence of noise trading and speculative bubbles is suspected.
**Neokosmidis** [30]	Six years’ data from March 2003 to March 2009 for four US stock indices i.e., Dow Jones, Nasdaq, NYSE, S&P500	ARCH, GARCH (1,1), EGARCH (1,1) Multivariate volatility models	The study concludes that EGARCH model is that best fitted process for all the sample data based on AIC minimum criterion. It is observed that there are high volatility periods at the beginning and at the end of our estimation period for all stock indices.
**Tripathy & Alana** [31]	Daily OHLC values of NSE index returns from 2005–2008	Rolling window moving average estimator, EWMA, GARCH models, Extreme value indicators, and Volatility index (VIX)	A GARCH and VIX models, proved to be the best methods. Extreme value models fail to perform because of low frequency data.
**Liu & Hung** [32]	Taiwanese stock index futures prices, daily data April 2001 to December 2008	GARCH type models: GARCH, GJR-GARCH, QGARCH, EGARCH, IGARCH, CGARCH	They demonstrate that the EGARCH model provides the most accurate daily volatility forecasts, while the performances of the standard GARCH model and the GARCH models with highly persistent and long-memory characteristics are relatively poor.
**Joshi** [33]	Daily closing price from January 2005 to May 2009	BDS Test, ARCH-LM test, and GARCH (1,1) model	Persistence of volatility is more than Indian stock market
**Wong & Cheung** [34]	Hong Kong stock market from 1984 to 2009	GARCH family models	The EGARCH and AGARCH models can detect the asymmetric effect well in response to both good news and bad news. By comparing different GARCH models, they find that it is the EGARCH model that best fits the Hong Kong case.
**Chang et al.** [35]	Taiwan Stock Exchange (TAIEX), the S&P 500 Index, and the Nasdaq Composite Index for the period of January, 2000 to January, 2004	GJR-GARCH model (1,1)	There is a significant price transmission effect and volatility asymmetry among the TAIEX, the US spot index, and US index futures.
**Koutmos** [36]	Shanghai stock exchange Ten industries sector indices daily data ranging from January 2009 to June 2012	Volatility estimation AR (1), EGARCH (1,1)	Time varying beta risk of industry sector indices in Shanghai stock results industries respond positively to rises in such non-diversifiable risk. Reports on the volatility persistence of the various industry sectors and identifies which industries have high and low persistence.
**Chen** [37]	New York, London and Tokyo as well as those of Hong Kong, Shanghai and Shenzen the period of January 1993 to March 2010	Granger causality test, VAR model, VEC model, variance decomposition, impulse response function, co-integration and GARCH models	Evidence shows that five stock markets are in the process of increasing integration. The periodic break down of co-integrating relationship is advantageous to foreign investors.
**Abdalla & Suliman** [38]	Saudi stock market by using (Tadawul All Share Index; TASI) over the period of January 2007 to November 2011	GARCH (1,1) model, including both symmetric and asymmetric models	The results provide evidence of the existence of a positive risk premium, which supports the positive correlation hypothesis between volatility and the expected stock returns.
**Maheshchandra** [39]	Daily closing price of BSE and NSE stock indices period of January 2008 to August 2011	ARFIMA and FIGARCH models	Absence of long memory in return series of the Indian stock market. Strong evidence of long memory in conditional variance of stock indices.
**Li & Wang** [40]	China stock indices, six industry indexes, January 2006 to June 2012	ARMA and GARCH family model, GARCH (1,1), TGARCH (1,1), EGARCH (1,1)	The paper examined the leverage effect and information symmetry. Both ARCH and GARCH models can explain volatility clustering phenomena and have been quite successful in modeling real data in various applications.
**Hou** [41]	Daily closing prices of the SHCI and SZCI indices from January 1997 to August 2007	GARCH family models	An asymmetric effect of negative news exists in the Chinese stock markets. The EGARCH and the GJR models tend to overestimate the volatility and returns in the high-volatility periods.
**Purohit et al.** [42]	Daily closing data for November 2009 to March 2013, NIFTY and NIFTY Junior indices	ADF Test, Johansen’s co-integration test, and GARCH (1,1) model	Empirical results found that one-month futures do not bring volatility in the VIX.
**Shalini** [43]	Daily data of sectoral indices for the period of January 2001 to June 2014	ARMA (1,1), and GARCH (1,1) models	Return of the BSE sectoral indices exhibit characteristics of normality, stationarity, and heteroscedasticity.
**Ghorbel & Attafi** [44]	MENA stock market indices of daily observations for the period January 2007 to March 2012	GARCH family models	MENA region’s markets are higher between extremes than between ordinary observations registered during normal periods, but they offer many opportunities to investors to diversify their portfolio and reduce their degree of risk aversion. Dependence between markets increases during volatile periods.
**Gupta et al.** [45]	The daily closing prices of S&P CNX500 of National Stock Exchange for the period from January 2003 to December 2012	GARCH, TGARCH, and EGARCH models	The result of that volatility varies over time and constant variance assumption is inconsistent. The empirical evidence indicated the presence of time varying volatility.
**Nadhem et al.** [46]	S&P500 market daily returns the sample period from July 1996 to May 2006	GARCH family models	Results of ANN models will be compared with time series model using GARCH family models. The use of the novel model for conditional stock markets returns volatility can handle the vast amount of nonlinear data, simulate their relationship, and give a moderate solution for the hard problem.
**Banumathy & Azhagaiah** [47]	The daily closing prices of S&P CNX Nifty Index for the period from January 2003 to December 2012	Both symmetric and asymmetric models GARCH (1,1)	The result proves that GARCH and TGARCH estimations are found to be the most appropriate model to capture symmetric and asymmetric volatility respectively.
**Okičić** [48]	Central and Eastern Europe region for the period from October 2005 to December 2013	Both symmetric and asymmetric GARCH models, i.e.,; GARCH, IGARCH, EGARCH, GJR, and PGARCH	Study indicate that existence of the leverage effect in case of stock markets from the CEE region, which indicates that negative shocks increase the volatility more than positive shocks.
**Lum & Islam** [49]	Australian share markets data for the period of January 1988 to December 2004	GARCH family models	Findings support asymmetric effects in the Australian share markets, and by incorporating them into the GARCH-M models yield better results in both financial and econometric terms.
**Jebran & Iqbal** [50]	Asian countries, i.e., Pakistan, India, Sri Lanka, China, Japan, and Hong Kong. The daily data was considered from the period January 1999 to January 2014	GARCH model	Result revealed absence of any spillover effect of volatility across Indian and Chinese stock markets. However, bidirectional and unidirectional spillover effects have been established across other Asian markets.
**Yang et al.** [51]	CSI 300 index consider for the period of July 2013 to January 2016	GARCH, EGARCH, APARCH, and PTTGARCH models	The PTTGARCH models both with single regime and Markov regime switching outperform other models in estimation and prediction of the volatilities of the return series within the sample and out-of-sample.
**Varughese & Mathew** [52]	India stock market daily data for the period of April 2003 to March 2015	GARCH, EGARCH, and TARCH models	The existence of volatility clustering and leverage effect in the market and the investment activities of foreign portfolio investment have had a significant impact on the volatility of stock market.
**Pati et al.** [53]	India NIFTY Volatility Index (IVIX) and CNX NIFTY Index (NIFTY), Australia S&P/ASX 200 Volatility Index (AVIX) and S&P/ASX 200 Index (ASX), and Hong Kong Hang Seng Volatility Index (VHSI) and HSI, consider the period of January 2008 to July 2016	GARCH family models	The study finds that volatility index is a biased forecast but possesses relevant information in explaining future realized volatility. GARCH family models suggest that it contains relevant information in describing the volatility process.
**Pele et al.** [54]	The EUR/JPY exchange rate of daily prices and time period considered from 1999 to 2005	GARCH model, Entropy, and VAR model	GARCH-based forecast is more stable whilst the entropy-based forecast reacts faster to new information. VAR model performs the worst failing the tests, whilst the normal GARCH model passes all tests. But the best results overall are obtained by the entropy-based forecast model.
**Bhowmik et al.** [55]	Emerging six Asian stock markets daily stock market index data from January 2002 to December 2016	GARCH model, Granger Causality Tests, and VAR model	The volatility and return spillovers behave very differently over time, during the pre-crisis, crisis, and post crisis periods. Importantly, the Asian emerging stock markets interaction was less before the global financial crisis period.
**Kim & Lee** [56]	Daily negative returns of the Google’s stock price and Dow Jones index, November 2004 to November 2016	PTTGARCH model	Article demonstrates its validity through a simulation study and real data analysis. The result indicates that for practical applications, the underlying innovation distribution should be modeled in a more refined manner.
**Amudha & Muthukamu** [57]	NSE from the period of April 2003 to September 2015	GARCH family models	The findings reported an evidence of volatility, which exhibited the clustering and persistence of stocks. The return series of the stocks selected for the study were found to react on good and bad news asymmetrically.
**Chronopoulos et al.** [58]	US stock return a daily frequency S&P500 index covering the period from January 2004 to December 2016	GARCH family models	The SVI variable exhibits the best performance among all considered models and SVI variable offers the highest gains for investors.
**Bhowmik & Wang** [59]	BSE 30, SSE composite, DSEX, FBMKLCI, PSEi, KOSPI indices data of daily closing prices for the period of January 2007 to 2016	GARCH family models and VAR model	The returns and volatility linkages exist between the emerging Asian markets and the developed stock markets. The volatilities to unexpected shocks in various markets, especially, come from neighboring country markets and more developed country markets.
**Kapusuzoglu & Ceylan** [60]	Borsa Istanbul sector indices of daily data over the period of October 1987 to January 2017	GARCH model	Model shows the existence of a positive and statistically significant relationships between trading volume and the number of information events makes the variability of the sector indices to increase.
**Wang et al.** [61]	High frequency data, stock market policies issued related news, January 2014 to August 2015	GARCH-M and EGARCH-M models	The results show that China’s stock market was mainly driven by government policies rather than economic fundamentals, as measured by GDP, PPI, and PMI.
**Shanthi & Thamilselvan** [62]	Nifty 50 and BSE Sensex daily data from both indices over the period of January 1995 to December 2015	GARCH, TGARCH, and EGARCH models	The study indicates that symmetric information is not suitable for a certain period considered in this study. The TGARCH model outperformed all the models due to the availability of information.
**Bhowmik & Wang** [63]	The data consists of daily, weekly, and monthly closing prices of six emerging stock market indexes in Asian countries from the period of 2007 to 2016	Unit root tests, serial correlation test, runs test, VR tests, ARMA, GARCH model, and BDS test	Study suggests that none of the sample Asian emerging stock markets follow Random-walk and hence all are weak-form efficient markets except South Korean Markets. Additionally, short-term variants of the technical trading rules have better predictive ability than long-term variants.
**Dixit & Agrawal** [64]	BSE and NSE daily data of the closing value from April 2011 to March 2017	GARCH family models	The study suggested that the P-GARCH model is most suitable to predict and forecast the stock market volatility for BSE and NSE markets.
**Kumar & Biswal** [65]	Brazil, India, Indonesia and Pakistan stock markets return of the average price (open, close, high, and low) for January 2014 to October 2018	GARCH family models	The result confirms the presence of volatility clustering and leverage effect that is that good news affects the future stock market than bad news.

Notes: APARCH (Asymmetric Power ARCH), AIC (Akaike Information Criterion), OHLC (Open-High-Low-Close Chart), NSE (National Stock Exchange of India), EWMA (Exponentially Weighted Moving Average), CGARCH (Component GARCH), BDS (Brock, Dechert & Scheinkman) Test, ARCH-LM (ARCH-Lagrange Multiplier) test, VAR (Vector Autoregression) model, VEC (Vector Error Correction) model, ARFIMA (Autoregressive Fractional Integral Moving Average), FIGARCH (Fractionally Integrated GARCH), SHCI (Shanghai Stock Exchange Composite Index), SZCI (Shenzhen Stock Exchange Component Index), ADF (Augmented Dickey–Fuller) test, BSE (Bombay Stock Exchange), and PGARCH (Periodic GARCH) are discussed.

Based on the researchers’ articles, the symmetric GARCH (1, 1) model has been used widely to forecast the unconditional volatility in the stock market and time series data, and has been able to simulate the asset yield structure and implied volatility structure. Most researchers show that GARCH (1, 1) with a generalized distribution of residual has more advantages in volatility assessment than other models. Conversely, the asymmetry influence in stock market volatility and return analysis was beyond the descriptive power of the asymmetric GARCH models, as the models could capture more specifics. Besides, the asymmetric GARCH models can incompletely measure the effect of positive or negative shocks in stock market return and volatility, and the GARCH (1, 1) comparatively failed to accomplish this fact. In asymmetric effect, the GJR-GARCH model performed better and produced a higher predictable conditional variance during the period of high volatility. In addition, among the asymmetric GARCH models, the reflection of EGARCH model appeared to be superior.

Table 4 has explained the review of bivariate and other multivariate GARCH models. Bivariate model analysis was used to find out if there is a relationship between two different variables. Bivariate model uses one dependent variable and one independent variable. Additionally, the Multivariate GARCH model is a model for two or more time series. Multivariate GARCH models are used to model for forecast volatility of several time series when there are some linkages between them. Multivariate model uses one dependent variable and more than one independent variable. In this case, the current volatility of one time series is influenced not only by its own past innovation, but also by past innovations to volatilities of other time series.

The most recognizable use of multivariate GARCH models is the analysis of the relations between the volatilities and co-volatilities of several markets. A multivariate model would create a more dependable model than separate univariate models. The vector error correction (VEC) models is the first MGARCH model which was introduced by Bollerslev et al. [66]. This model is typically related to subsequent formulations. The model can be expressed in the following form: $vech\;(H_{t})=\mathbb{C}+\sum_{j=1}^{q}X_{j}\;vech\;\left(\epsilon_{t-j}\;\epsilon_{t-j}^{'}\right)+\sum_{j=1}^{p}Y_{j}\;vech\;\left(H_{t-j}\;\right)\;$where vech is an operator that stacks the columns of the lower triangular part of its argument square matrix and $H_{t}$ is the covariance matrix of the residuals. The regulated version of the VEC model is the DVEC model and was also recommended by Bollerslev et al. [66]. Compared to the VEC model, the estimation method proceeded far more smoothly in the DVEC model. The Baba-Engle-Kraft-Kroner (BEKK) model was introduced by Baba et al. [67] and is an innovative parameterization of the conditional variance matrix $H_{t}$. The BEKK model accomplishes the positive assurance of the conditional covariance by conveying the model in a way that this property is implied by the model structure. The Constant Conditional Correlation (CCC) model was recommended by Bollerslev [68], to primarily model the conditional covariance matrix circuitously by estimating the conditional correlation matrix. The Dynamic Conditional Correlation (DCC) model was introduced by Engle [69] and is a nonlinear mixture of univariate GARCH models and also a generalized variety of the CCC model. To overcome the inconveniency of huge number of parameters, the O-GARCH model was recommended by Alexander and Chibumba [70] and consequently developed by Alexander [71,72]. Furthermore, a multivariate GARCH model GO-GARCH model was introduced by Bauwens et al. [73].

**Table 4:** Different literature studies based on bivariate and other multivariate GARCH models.

Authors	Data Set	Econometric Models	Study Results
**Singh et al.** [74]	15 world indices for the period of January 2000 to February 2008 have been considered	AR-GARCH, bivariate VAR, Multivariate GARCH (BEKK) model	There is significant positive volatility spillover from other markets to Indian market, mainly from Hong Kong, Korea, Japan, and Singapore and US market. Indian market affects negatively the volatility of US and Pakistan.
**Rao** [75]	Daily returns data from February 2003 to January 2006, Arabian Gulf Cooperation Council equity markets data	MGARCH and VAR models	Arabian Gulf Cooperation Council markets exhibit significant own and cross spillover of innovations and volatility spillover and persistence in these markets.
**Maniya & Magnnsson** [76]	S&P 500, NIKKE 225, KSE 100, BSE 30, Hang Seng indices. Daily closing Index and data from January 1989 to December 2009	ARCH, GARCH models, GARCH-BEKK model correlation, unit root tests, granger-causality test	Time varying correlation increases in bearish spells whereas bullish periods do not have a big “Statistical” impact on correlation.
**Princ [77]**	Daily returns of Prague stock exchange index and other 11 major stock indices during 1994 to 2009	DCC-MVGARCH model	The study found the existence of an increasing trend in conditional correlations among a whole European region. Results show the unidirectional influence of foreign markets affecting Czech market.
**Yong et al.** [78]	Daily data of Japanese stock over the study period 1994–2007	BEKK-GARCH model	They found that news shocks in the Japanese currency market account for volatility transmission in eight of the 10 industrial sectors considered. They also found that significant asymmetric effects in five of these industries.
**Athukoralalage** [79]	Weekly stock market data of Australia, Singapore, UK, and the US for the period from Jan 1992 to June 2010	M-GARCH Model, Diagonal BEKK model ARCH, and GARCH techniques	Positive return spillover effects are only unidirectional and run from both US and UK (the bigger markets) to Australia and Singapore (the smaller markets). Shocks arising from the US market can impact on all of the other markets in the sample.
**Kouki et al.** [80]	Five sectors daily data covering period from January 2002 to October 2009	VAR Framework one lag, BEKK (1,1) model	International financial markets are not integrated in all the sectors. Results find that the three highly integrated sectors are bank, real estate, and oil.
**Walid et al.** [81]	The weekly closing stock indexes and local currency and exchange rates used for four emerging markets, data from December 1994 to March 2009	Markov-Switching-EGARCH model	Results provide strong evidence that the relationship between stock and foreign exchange market is regime dependent and stock price volatility responds asymmetrically to events in the foreign exchange market.
**Katzke [82]**	Daily closing prices of six largest industrial sector composite total return indices during January 2002 to April 2013	AR (1) model, MV-GARCH models, DCC models, VECH, and BEKK techniques, and GJR-GARCH model	The results show that global and domestic economic uncertainty as well as local asset market segment significantly influences both the short run dynamics and the aggregate level of co-movement between local sector pairs.
**Peng et al.** [83]	TAIEX and Nikkei from both indices over the period of January, 2000 to March, 2016	Bi-EGARCH model	The past returns on NIKKEI influenced significantly current period returns of TAIEX, yet there was no such influence flowing from past returns of TAIEX to the current returns on NIKKEI index. Furthermore, the two stock markets are more sensitive to falling rather than rising trends of each other, implying that there is a mutual tendency between these markets to crash due to a retreat in the counterpart market.
**Lv et al.** [84]	GEM index china, daily return data over the period of January 2014 to June 2018	DCC-MV-GARCH model, bivariate EGARCH model and VECM model	The network entropy indices increased in the period of the market crash. Equity market-trading activity and network entropy were informationally efficient in the long run and the more heterogeneous the stock network is, the higher market returns.

The bivariate models showed achieve better in most cases, compared with the univariate models [85]. MGARCH models could be used for forecasting. Multivariate GARCH modeling delivered a realistic but parsimonious measurement of the variance matrix, confirming its positivity. However, by analyzing the relative forecasting accuracy of the two formulations, BEKK and DCC, it could be deduced that the forecasting performance of the MGARCH models was not always satisfactory. By comparing it with the other multivariate GARCH models, BEKK-GARCH model was comparatively better and flexible but it needed too many parameters for multiple time series. Conversely, for the area of forecasting, the DCC-GARCH model was more parsimonious. In this regard, it was significantly essential to balance parsimony and flexibility when modeling multivariate GARCH models.

The current systematic review has identified 50 research articles for studies on significant aspects of stock market return and volatility, review types, and GARCH model analysis. This paper noticed that all the studies in this review used an investigational research method. A literature review is necessary for scholars, academics, and practitioners. However, assessing various kinds of literature reviews can be challenging. There is no use for outstanding and demanding literature review articles, since if they do not provide a sufficient contribution and something that is recent, it will not be published. Too often, literature reviews are fairly descriptive overviews of research carried out among particular years that draw data on the number of articles published, subject matter covered, authors represented, and maybe methods used, without conducting a deeper investigation. However, conducting a literature review and examining its standard can be challenging, for this reason, this article provides some rigorous literature reviews and, in the long run, to provide better research.

## 4. Conclusions

Working on a literature review is a challenge. This paper presents a comprehensive literature which has mainly focused on studies on return and volatility of stock market using systematic review methods on various financial markets around the world. This review was driven by researchers’ available recommendations for accompanying systematic literature reviews to search, examine, and categorize all existing and accessible literature on market volatility and returns [16]. Out of the 435 initial research articles located in renowned electronic databases, 50 appropriate research articles were extracted through cross-reference snowballing. These research articles were evaluated for the quality of proof they produced and were further examined. The raw data were offered by the authors from the literature together with explanations of the data and key fundamental concepts. The outcomes, in this research, delivered future magnitudes to research experts for further work on the return and volatility of stock market.

Stock market return and volatility analysis is a relatively important and emerging field of research. There has been plenty of research on financial market volatility and return because of easily increasing accessibility and availability of researchable data and computing capability. The GARCH type models have a good model on stock market volatilities and returns investigation. The popularity of various GARCH family models has increased in recent times. Every model has its specific strengths and weaknesses and has at influence such a large number of GARCH models. To sum up the reviewed papers, many scholars suggest that the GARCH family model provides better results combined with another statistical technique. Based on the study, much of the research showed that with symmetric information, GARCH (1, 1) could precisely explain the volatilities and returns of the data and when under conditions of asymmetric information, the asymmetric GARCH models would be more appropriate [7,32,40,47,48]. Additionally, few researchers have used multivariate GARCH model statistical techniques for analyzing market volatility and returns to show that a more accurate and better results can be found by multivariate GARCH family models. Asymmetric GARCH models, for instance and like, EGARCH, GJR GARCH, and TGARCH, etc. have been introduced to capture the effect of bad news on the change in volatility of stock returns [42,58,62]. This study, although short and particular, attempted to give the scholar a concept of different methods found in this systematic literature review.

With respect to assessing scholars’ articles, the finding was that rankings and specifically only one GARCH model was sensitive to the different stock market volatilities and returns analysis, because the stock market does not have similar characteristics. For this reason, the stock market and model choice are little bit difficult and display little sensitivity to the ranking criterion and estimation methodology, additionally applying software is also another matter. The key challenge for researchers is finding the characteristics in stock market summarization using different kinds of local stock market returns, volatility detection, world stock market volatility, returns, and other data. Additional challenges are modeled by differences of expression between different languages. From an investigation perception, it has been detected that different authors and researchers use special datasets for the valuation of their methods, which may put boundary assessments between research papers.

Whenever there is assurance that scholars build on high accuracy, it will be easier to recognize genuine research gaps instead of merely conducting the same research again and again, so as to progress better and create more appropriate hypotheses and research questions, and, consequently, to raise the standard of research for future generation. This study will be beneficial for researchers, scholars, stock exchanges, regulators, governments, investors, and other concerned parties. The current study also contributes to the scope of further research in the area of stock volatility and returns. The content analysis can be executed taking the literature of the last few decades. It determined that a lot of methodologies like GARCH models, Johansen models, VECM, Impulse response functions, and Granger causality tests are practiced broadly in examining stock market volatility and return analysis across countries as well as among sectors with in a country.

## Figures and Tables

**Table 1:** Literature search strings for database.

Search String	Search Field	Number of Non-Exclusive Results		
		Scopus	Web-of-Science	Last Updated
Market Return	Title/Article title	1540	1148	17 January 2020
Market volatility	Topic/Article title, Abstract, Keywords	13,892	13,767	17 January 2020
Stock market return	Topic/Article title, Abstract, Keywords	11,567	13,440	17 January 2020
Stock market volatility	Topic/Article title, Abstract, Keywords	5683	6853	17 January 2020
Market return and volatility	Topic/Article title, Abstract, Keywords	3241	6632	17 January 2020
GARCH family model* for stock return	Topic/Article title, Abstract, Keywords	53	41	17 January 2020
Forecasting stock return and GARCH model*	Topic/Article title, Abstract, Keywords	227	349	17 January 2020
Financial market return and volatility	Topic/Article title, Abstract, Keywords	2212	2638	17 January 2020

**Table 2:** Affiliation criteria.

Affiliation Criteria	Rational Explanation
Abstract must express the stock market and GARCH model as the sharp object of this research work.	Since this kind of research is not restricted to any journals, research on other subjects than stock market maybe appears.
Abstract must show clear indication of stock market volatility and return studies through GARCH model robustness.	The focus of the research is to study stock market return and volatility analysis by GARCH family model.
Research paper must be written in English language.	English language is the leading research language in the arena of finance.
